# Multicomponent Magneto-Orbital Order and Magneto-Orbitons in Monolayer VCl_3_

**DOI:** 10.1021/acs.nanolett.4c06400

**Published:** 2025-02-17

**Authors:** Luigi Camerano, Adolfo O. Fumega, Gianni Profeta, Jose L. Lado

**Affiliations:** † Department of Physical and Chemical Sciences, University of L’Aquila, Via Vetoio, 67100 L’Aquila, Italy; ‡ Department of Applied Physics, Aalto University, 02150 Espoo, Finland; § CNR-SPIN L’Aquila, Via Vetoio, 67100 L’Aquila, Italy

**Keywords:** Orbital ordering, Magnetism, Multicomponent order, 2D materials, Magneto-orbitons, Hybrid excitations

## Abstract

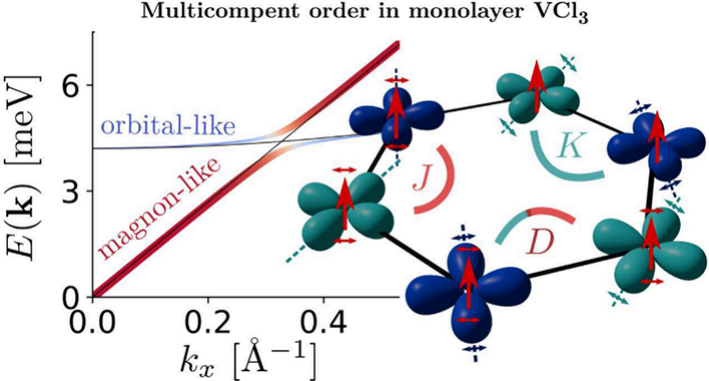

Van der Waals monolayers featuring magnetic states provide fundamental building blocks for artificial quantum matter. Here, we establish the emergence of a multicomponent ground state featuring magneto-orbital excitations of the 3*d*^2^-transition metal trihalide VCl_3_ monolayer. We show that monolayer VCl_3_ realizes a ground state with simultaneous magnetic and orbital ordering by using density functional theory. Using first-principles methods we derive an effective Hamiltonian with intertwined spin and orbital degrees of freedom, which we demonstrate can be tuned by strain. We show that magneto-orbitons appear as the collective modes of this complex order and arise from coupled orbiton magnon excitations due to the magneto-orbital coupling in the system. Our results establish VCl_3_ is a promising 2D material to observe emergent magneto-orbital excitations and provides a platform for multicomponent symmetry breaking.

Correlations in van der Waals materials represent the driving force behind a variety of unconventional electronic states, including unconventional superconductivity,^1−3^ exotic magnetism,^4^ and fractional topological states.^5,6^ Van der Waals magnetic materials^7,8^ provide a highly tunable platform to engineer a variety of quantum states, including ferromagnetism in CrBr_3_ and CrI_3_,^9,10^ antiferromagnetism in FePS_3_,^11^ heavy-fermion Kondo states in CeSiI^12,13^ and in dichalcogenide bilayers,^14−16^ quantum spin liquid candidates in RuCl_3_,^17,18^ 1T-TaSe_2_,^19^ and NbSe_2_,^20^ orbital magnets in twisted graphene bilayers^4,21^ and dichalcogenide bilayers,^6,22^ and multiferroic order in NiI_2_^23,24^ and twisted CrBr_3_ bilayers.^25−28^ Among them, multiferroic materials feature, besides a magnetic order in the spin degree of freedom, an additional electronic ordering in a spatial degree of freedom. While multiferroic monolayers such as NiI_2_^23,24,29−32^ feature simultaneous magnetism and ferroelectricity, a variety of other multicomponent orders are potentially possible, in particular associated with an electronic reorganization in an internal orbital degree of freedom.^33^ Among van der Waals magnets, VCl_3_ provides an ideal playground for complex electronic ordering due to the existence of orbital degeneracy leading to different potentially competing ground states.^34^ VCl_3_ belongs to family of vanadium trihalide materials, magnetic insulators due to its partially filled d-shell and strong correlations.^35−41^ Orbital ordering in monolayers provides a unique platform to stabilize exotic multicomponent orders tunable via substrate,^42^ electric gate,^43^ or twist engineering, in contrast with the more challenging control of orbital order in bulk compounds.^33,44−47^ VCl_3_ is an ideal candidate for orbital order due to its weaker spin–orbit coupling, which often quenches the orbital order in heavier halides.

Here, we establish emergence of a multicomponent ordering in VCl_3_, featuring anti-ferro-orbital ordering coupled to a coexisting magnetic phase. Using first-principles methods, we show that the orbital degeneracy in VCl_3_ gives rise to different magnetic and orbital orderings, with the lowest energy configuration tunable by an external strain. We further show that these two orders are not independent but strongly coupled, leading to strong magneto-orbital effects. This coupling gives rise to the appearance of new hybrid quasiparticles emerging from magnon and orbital excitations. Our results establish VCl_3_ as a paradigmatic material to realize multiferroic orbital ordering, providing a van der Waals monolayer enabling the observation magneto-orbital excitations.

We first addressed the electronic structure of VCl_3_ in the monolayer limit. VCl_3_ hosts 2 electrons in the 3-fold *t*_2*g*_ manifold in an octahedral environment *O*_*h*_ (see Figure 1a) similar to those observed in ABO_3_ perovskites.^44−47^ Due to partial occupation of *d* orbitals in the *t*_2*g*_ shell, a Jahn–Teller distortion^48^ of the octahedra lowers the symmetry from *O*_*h*_ to trigonal point group *D*_3*d*_ (see Figure 1a), splitting the *t*_2*g*_ manifold in a singlet *a*_1*g*_ and a doublet *e*_*g*_^′^. Spontaneous symmetry breaking leads to a further splitting of the *e*_*g*_^′^ manifold, lifting the orbital degeneracy. The occupation of these two nearly degenerate states for each V atom gives rise to orbital ordered phases, which can have a ferro-orbital (FO) (all the V atoms are in the same orbital configuration) or anti-ferro-orbital (AFO) (nearest-neighbor V atoms are in different orbital configuration) phases. The first-principles DFT+*U* method (*U* = 3.2 eV) yields symmetry broken orbitally ordered phases, always favored in energy with respect to both the *a*_1*g*_^1^*e*_*g*_^′1^ metallic phase and the *e*_*g*_^′2^ insulating phase. These orbitally ordered phases are stabilized by strong correlation, as clear from their evolution as a function of the *U* parameter. As DFT calculations feature a competition between electronic and magnetic degrees of freedom,^34^ the convergence of the orbital ordered phases in a DFT+*U* framework is particularly challenging and requires a symmetry-unconstrained unit cell and the use of the *d*-density matrix occupation control. By its suitable initial guesses, we compute both FO and AFO ordered phases, whose magnetization densities are reported in Figure 1b. In the case of orbital ordered phases, the electronic instability is followed by a different lowering of the crystal symmetry (from *D*_3*d*_ to *C*_2*h*_ for the FO and to *C*_1*h*_ for the AFO). It is worth noting how in the case of AFO the inversion symmetry of the honeycomb lattice is broken, possibly inducing a nonzero charge polarization. Comparing the energy of the FO and AFO, we find that the AFO phase is favored, meaning that the ground state of the system is the AFO ferromagnetic phase. In order to study the stability of this solution for a wide range of structural parameters, we applied in-plane strain to the lattice, finding that the AFO phase always remains stable. The orbital ordering mechanism can be directly visualized from the density of states on vanadium *d*_*z*^2^_, *d*_*xy*_, and showing the different occupation of the in-plane *d*_*xy*_ and  orbitals in the AFO phase but the same occupation of the *d*_*z*^2^_ orbital (the *d*_*z*^2^_ in global coordinates is the *a*_1*g*_ orbital in a trigonal symmetry, while the *d*_*xy*_ ∼ *e*_*g*,1_^′^ and  orbitals are a good representation of the *e*_*g*_^′^ manifold). The orbitally ordered phases in a magnetic van der Waals material give rise to a multicomponent ordering, which, if coupled, can further stabilize composite excitations. We address this possibility by deriving a model Hamiltonian from the converged first principle phases, using a spin S⃗ and pseudospin τ^*z*^ (describing the orbital configuration) operators to uncover the possible interaction between spin and orbital degree of freedom.

**Figure 1:**
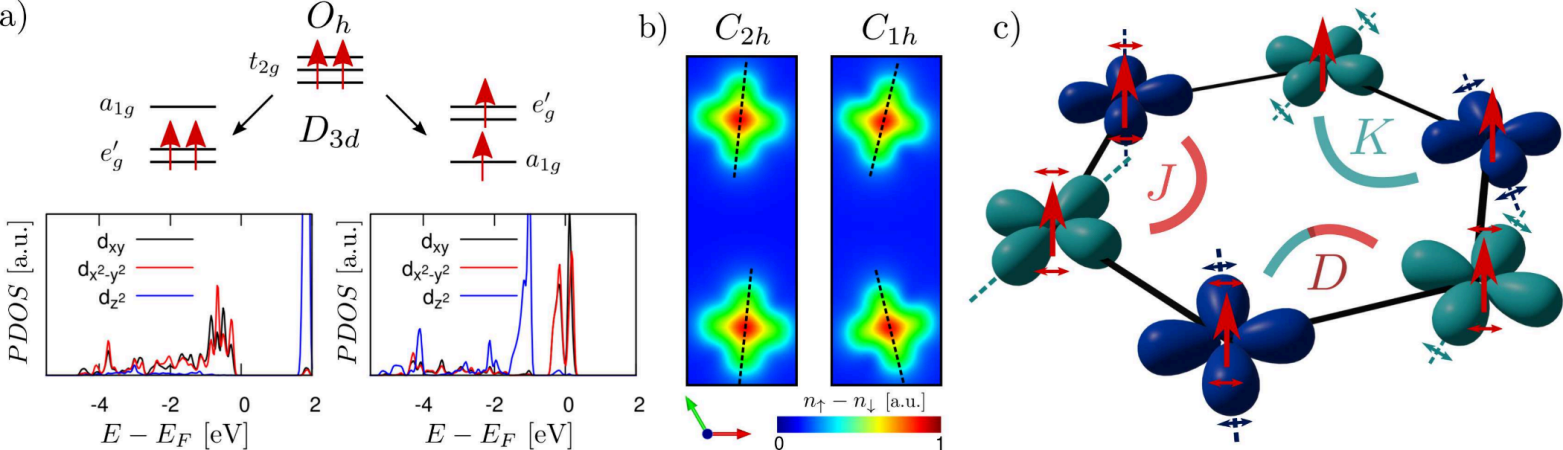
a) Schematic representation of the V-*d* orbitals in an octahedral environment (*O*_*h*_) and two possible electronic configurations after trigonal distortion (*D*_3*d*_). The corresponding DFT+*U* density of state projected on V-*d* is reported in the lower panel, showing orbital order states. b) First-principles calculated magnetization density (*n*_*↑*_ – *n*_*↓*_) in the case of a ferromagnetic ferro-orbital (*C*_2*h*_ symmetry) and ferromagnetic anti-ferro-orbital (*C*_1*h*_ symmetry). c) Sketch of the model presented in this study; the different colors for the orbitals highlight different V-sites in the anti-ferro-orbital phase. The coupling constants *J*, *K*, and *D* refer to the model in eq 1.

The multiple degrees of freedom render VCl_3_ a material realizing an effective *SU*(4) = *SU*(2)_spin_ × *SU*(2)_orbital_ model, similar to flat bands of twisted graphene multilayers.^49−55^ In the orbital degree of freedom, *SU*(2)_orbital_ symmetry is broken into a *U*(1)_orbital_ symmetry due to the crystal lattice, in analogy with the valley degree of freedom in twisted graphene multilayers. Using first-principles calculations we can map the effective Hamiltonian in the spin and orbital degrees of freedom,^56^ which takes the form
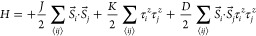
1where ⟨⟩ denotes first vanadium neighbors, *J* is the isotropic Heisenberg-like coupling, *K* is the anisotropic Ising-like coupling between pseudospins, and *D* represents the coupling between the spin and pseudospin variables. Since VCl_3_ hosts two electrons in a high spin configuration, *S* = 1, the pseudospin can be described by an Ising coupling with . Thus, *K* captures the orbital exchange interaction, while *D* the coupling with the spin degree of freedom. The schematic of this model is shown in Figure 1c. We take in eq 1 that there is a strong easy axis for the pseudospin associated with the explicit breaking of *SU*(2)_orbital_ due to the lattice. This assumption is justified by our first-principles calculation, which converges to the same orbital configuration shown in Figure 1b, even when the *d*-density matrix is initialized with rotated axes. Depending on the values of *J*, *K*, and *D*, different spin–orbital orders emerge from this model.^57−59^ By ab initio calculation, we calculated the effective parameters present in the Hamiltonian (1) through the stabilization of four possible ground states: (I) FO-FM, (II) FO-AFM, (III) AFO-FM, (IV) AFO-AFM (see Figure 2a), where FM and AFM stand for ferromagnetic and antiferromagnetic, respectively. We treat eq 1 in the classical approximation, i.e., describing spins S⃗ as dimensionless classical vectors of length *S* in the sphere and considering τ_*i*_^*z*^τ_*j*_^*z*^ = ±1. We denote the corresponding ground state energies as ϵ_*FO*,*FM*_, ϵ_*FO*,*AFM*_, ϵ_*AFO,FM*_, and ϵ_*AFO,AFM*_. The spin–orbital model allows us to write the energy per unit cell (honeycomb lattice 2 V atoms) of the different configurations as (we only write down the FO-FM phase for the sake of brevity) ϵ_*FO*,*FM*_ = +3*JS*^2^ + 3*K*^2^ + 3*D*^2^*S*^2^ + *E*_0_, where *E*_0_ is a constant energy term. In order to determine *J*, *K*, and *D*, we use the ground state energies for these 4 configurations as obtained from our DFT calculations. The obtained results are reported in Figure 2b–d as a function of the lattice parameter *a* and for different values of the Hubbard *U*. We note that the theoretical lattice constant at the DFT+*U* level is *a* = 6.24 Å, to be compared with the available experimental lattice parameter for the bulk, which is *a* = 6.01 Å.^60^ In general, we find *J* > *K* ≫ *D*; however, depending on the strain, the spin–orbital coupling *D* can be enhanced by 1 order of magnitude.

**Figure 2:**
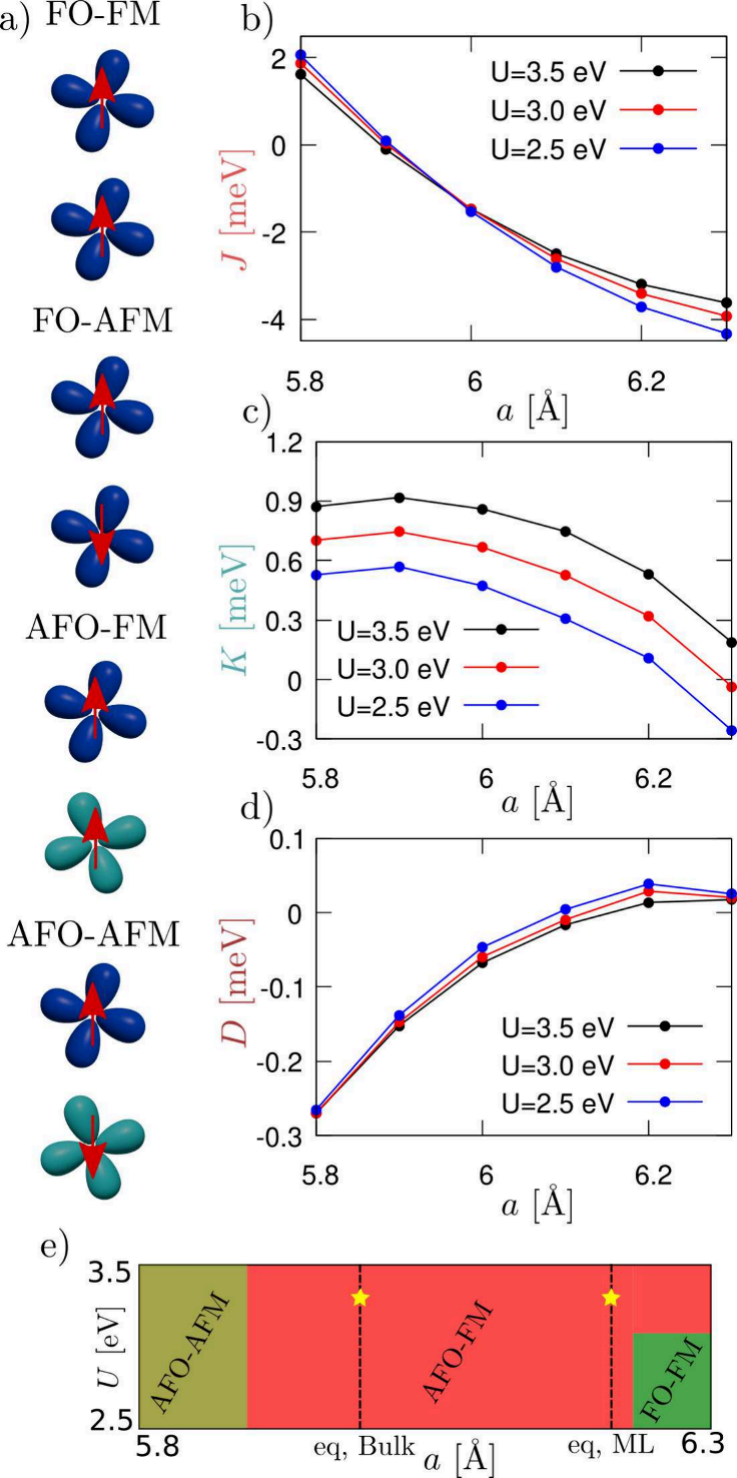
a) Schematic representation of the 4 states (see the text) that are needed to calculate the exchange coupling of the model. b–d) Isotropic Heisenberg coupling (*J*), anisotropic orbital exchange coupling (*K*), and spin–orbital coupling (*D*) as a function of the lattice parameter *a* for different values of the Hubbard repulsion *U*. e) A sketch of the phase diagram of the system as a function of strain and *U*; the yellow stars highlight the linear response calculated *U* value,^61^ and ML stands for monolayer.

With *a* = 5.80 Å, close to the experimental bulk lattice parameter, *D* = −0.27 meV independently of the *U* value. It is worth noting that the exchange orbital coupling *K* is highly dependent on the value of *U*. This aligns with the scenario in which correlation effects drive the orbital ordering. Specifically, higher *U* values result in stronger orbital coupling, while in the limit of small *U*, the orbital coupling is quenched. Another important feature is the dependence of the spin exchange coupling *J* on lattice strain, where at some strains the coupling switches from ferromagnetic to antiferromagnetic. This phenomenology may account for the significant impact of the substrate on the magnetic order, as recently demonstrated in ref (42). This allows for strain engineering of the magnetism, which in this material is coupled with the orbital and, consequently, the charge degrees of freedom. Finally, we note how for *a* = 5.9 Å, *J* is almost quenched while *D* is enhanced. In this regime, controlling the orbital configuration permits the switch from the ferromagnetic to the antiferromagnetic phase and vice versa. Thus, the first-principles mapping on the Hamiltonian in eq 1 reveals a sizable coupling between spin and orbital degrees of freedom in VCl_3_. To summarize the consequences of the values of *J*, *K*, and *D*, in panel (e) of Figure 2, we report a phase diagram of the system.

The magnetic and orbital order breaking gives rise to magnetic and orbital excitations which can be studied using the Hamiltonian in eq 1. Considering only the spin degree of freedom, gapless magnons will arise due to the continuous symmetry of the spin sector. Due to the 2D nature of the magnetism, magnetic anisotropy is necessary for magnetic order to occur. We do not include it in our model, as it does not influence the magnon dispersion aside from opening a small magnon gap.^10^ Orbital excitations will have gapped spectra due to the Ising-like interaction considered. To study elementary excitation, we can consider Hamiltonian (1) as composed of *H* = *H*_*s*_ + *H*_*o*_ + *H*_*int*_, where *H*_*s*_ is the only spin Hamiltonian, *H*_*o*_ the orbital one, and *H*_*int*_ is the interaction term proportional to *D*. *H*_*s*_ and *H*_*o*_ are studied in terms of linear spin waves (LSW) and linear orbital waves (LOW).^62−64^ We perform first a Holstein–Primakoff transformation introducing bosonic operators *b*_*i*_ and α_*i*_ (τ_*i*_^*z*^ = τ^*z*^ – *b*_*i*_^†^*b*_*i*_, *S*_*i*_^*z*^ = *S* – α_*i*_^†^α_*i*_, ,  for the ferromagnetic case); then a Bogoliubov transformation gives the magnon dispersion and introduces magnon creation and annihilation operators *a*_*i*_^†^ and *a*_*i*_. When the spin–orbital coupling is considered via *H*_*int*_, the full Hamiltonian *H* becomes biquadratic and a decoupling^65,66^ is performed. By introducing a spin–orbital hybridization function and neglecting the anomalous terms, we can write down the effective Hamiltonian *H*_*eff*_ as
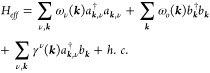
2where *a*_***k***,ν_^†^ and *b*_***k***_^†^ are bosonic operators that create a magnon with energy ω_ν_(**k**) in the magnon band ν and an orbiton with energy ω_*o*_(**k**), respectively. The spin–orbital hybridization function depends on the magneto-orbital coupling as γ^ν^(***k***) ∼ *D*⟨*a*_***k***_*b*_***k***_^†^⟩. ω_*o*_(**k**) is analogous to ω_ν_(**k**) for the magnons, but in our Hamiltonian (1), due to the Ising-like interaction between pseudospins, ω_*o*_(**k**) = 6*K*. If we consider the possibility of orbital in-plane rotation or a bond-dependent coupling, ω_*o*_(**k**) could depend on **k**, resulting in an orbital excitation dispersion. Finally, the magneto-orbital coupling also renormalizes the exchange spin and orbital interaction, as shown by rewriting eq 1 in the following form:  where *J*_*ij*_ = *J*_*ij*_(τ_*i*_^*z*^,τ_*j*_^*z*^).^67^

The Hamiltonian (2) is solved by expanding the Hilbert space of the spin states (first term in eq 2) to include the pseudospins (second term in (2)) and their hybridization with spins (third term in (2)). Since *J* > *K*, we expand the magnon and orbital dispersion near the Γ point. In this limit, the ferromagnetic dispersion is parabolic with an effective mass , while antiferromagnetic dispersion is linear with an effective velocity  in an honeycomb lattice. The results of the entangled magnon and orbital spectra are summarized in Figure 3 as a function of the strain. We first consider the case of the Hamiltonian (1), in which orbitals cannot rotate (Figure 3a–c). In the absence of spin–orbital hybridization (*D* = 0), the spectra consist of two different dispersions, ω_ν_(**k**) and ω_*o*_(**k**), which do not interact. In the presence of magneto-orbital coupling (*D* ≠ 0), a gap appears in the magnon spectrum with its amplitude proportional to the coupling. Moreover, magnons and orbitons hybridize at the crossing points, opening a gap giving rise to magnon-orbiton excitations (Figure 3). Thus, the multicomponent order gives rise to both orbiton excitations like the one detected in ref (68) and hybridized magnon-orbiton excitations which, to the best of our knowledge, have never been experimentally observed. In Figure 3d–f we consider the general case in which the interaction between orbitals is of the type ; that is, the pseudospin interaction allows both in-plane rotation and bond-dependent (γ) coupling. This leads to the emergence of an orbital dispersion ω_*o*_(**k**).^65−67^ In this case, the dispersion is not linear due to the strong anisotropy that describes the pseudospin variable. In order to verify if the energy gap is preserved including orbital dispersion, we consider an orbiton dispersion with an effective mass *m*_*eff,O*_ = *K*_⊥_τ^*z*^*a*^2^ where  as estimated in ref (67). The main consequence of ω_*o*_(**k**) is the tilting of the magnon bands at the points where the magnon and orbital bands intersect, still showing the presence of a gap. This effect becomes more pronounced as the orbital coupling increases (Figure 3d). Another consequence of the general pseudospin interaction, which we do not explore in this work but is worth mentioning, is the emergence of a Kitaev-like interaction between spins in the limit of strong SOC.^69,70^ This is not the case for VCl_3_ due to the lighter halide, but it could be the case for VI_3_ if entangled magneto-orbital ordered phases can also be stabilized in this compound.^71^ Finally, we show how the amplitude of the gap and the strength of the hybridization can be tuned by the in-plane strain. In particular, for a relatively large 6% compressive strain (Figure 3a–d) the system is an antiferromagnet with a very large magneto-orbital coupling (*D* = −0.27 meV) due to the increased hybridization, but only a 0.2% tensile strain, strongly decreasing the orbital exchange coupling and eventually causing it to disappear (for *a* > 6.4 Å our first-principles calculation cannot converge both FO and AFO phases).

**Figure 3:**
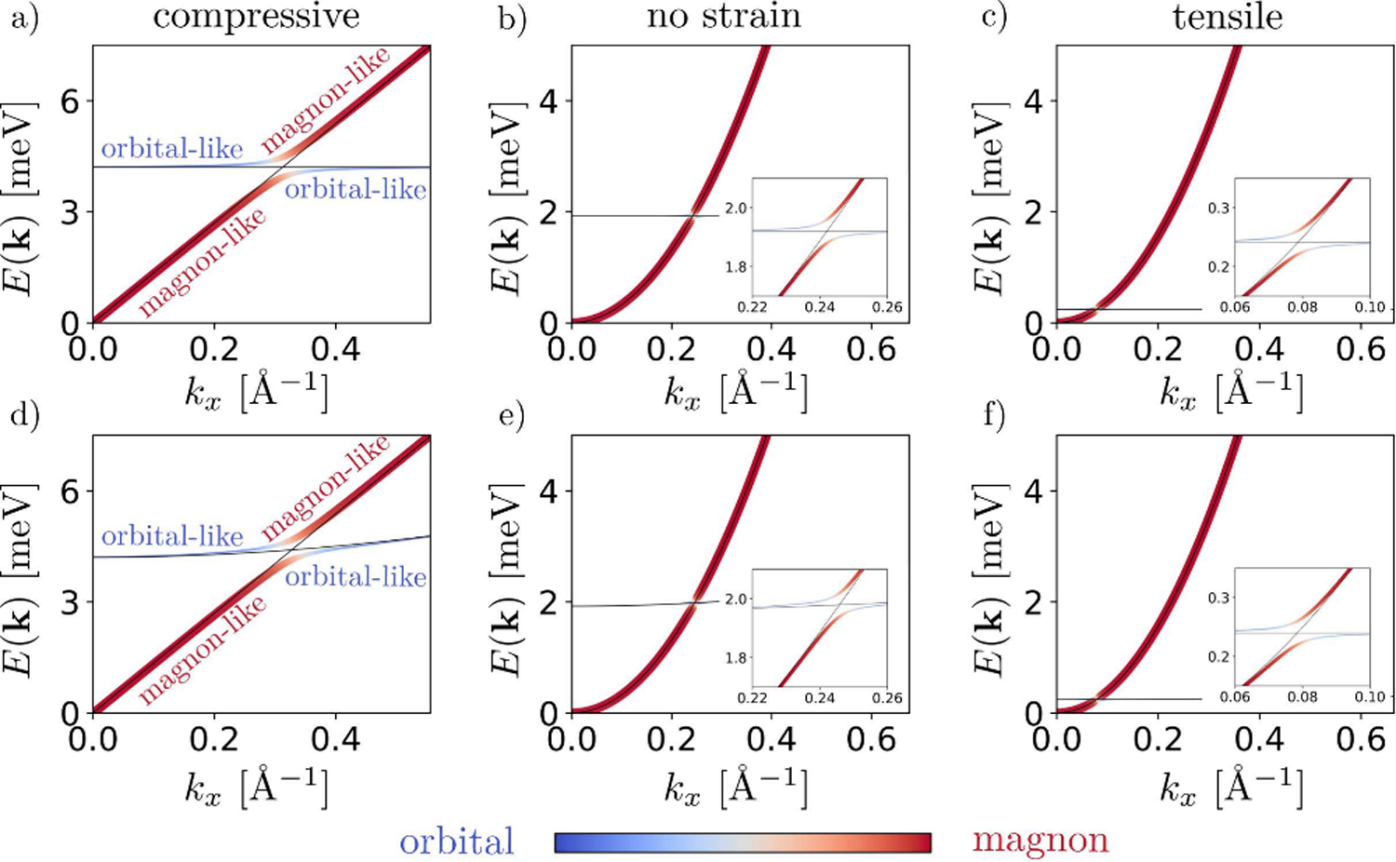
Magnon-orbital excitation as a function of the strain. In particular in a), b), and c) the excitation spectra are reported for the coupling values reported in Figure 2. In d), e), and f) a **k** dispersion for the orbital excitation is assumed (ω_*o*_(**k**)), representing the coupling with other orbitals (see text). The color bar represents the excitation character, and the line width is proportional to the projection on the magnon manifold. The black thin lines represent the magnon and orbital dispersion without hybridization (*D* = 0).

Finally, we briefly discuss some experimental techniques to probe our prediction. Various techniques offer complementary ways to analyze low-energy excitations, orbital ordering, and symmetry breaking. For example, STM measurements can provide detailed visualization of orbital ordering,^72^ while optical spectroscopy is effective in exciting low-energy states near Γ.^68^ Second-harmonic generation offers a valuable means to detect symmetry breaking associated with orbital ordering,^73^ and inelastic tunneling junctions^74^ allow simultaneous measurement of magnon and orbital excitations near the zone center. Neutron inelastic scattering would provide access to hybrid magneto-orbital excitations across the entire *k*-space.^75^

Here we show that VCl_3_ develops multiferroic order in both the orbital and spin degrees of freedom, giving rise to intertwined magnon and orbital excitations. Through first-principles calculations, we establish the appearance of strain-dependent ferro- and anti-ferro-orbital ordered phases in VCl_3_, coexisting with the magnetic state. The autonomous emergence of these orders classifies it as type-I multiferroic. This contrasts with monolayer NiI_2_, a paradigmatic example of type-II 2D multiferroics, characterized by strong magnetoelectric coupling. VCl_3_, therefore, represents the first example of a type-I 2D multiferroic, due to the weaker coupling (*J* ≫ *K* > *D*) between orders. Based on first-principles methods, we derived a low-energy Hamiltonian that accounts for the magnon and orbital excitations, including the effect of magneto-orbital coupling between both degrees of freedom. The magneto-orbital coupling gives rise to magneto-orbiton excitations stemming from the multicomponent order, establishing an exotic excitation that can be probed in this monolayer material. We show that magneto-orbital coupling, orbital order, and magnetic order are tunable with strain, making VCl_3_ a potential platform for magneto-orbital straintronics. In particular, inhomogeneous strain, such as that present in twisted heterostructures, is expected to give rise to moiré domains with different magneto-orbital ordering and magneto-orbital dynamics, providing a new playground in moiré matter. Our results establish the deep interplay between orbital and magnetic ordering in VCl_3_, presenting a paradigmatic example of a multicomponent ordered phase in van der Waals materials.

## Supporting Information Available

Computational methods; discussion of correlation effects in stabilizing orbital ordering, comparison between FO and AFO energies, projected density of state in both FO and AFO phases (PDF)
