# Supplementary material for: Multicomponent Magneto-Orbital Order and Magneto-Orbitons in Monolayer VCl3
Source: Nano Lett. 2025 Feb 17;25(12):4825–31. doi: 10.1021/acs.nanolett.4c06400 (PMC11951159; doi:10.1021/acs.nanolett.4c06400)
Supplement: Supplementary file 1 — nl4c06400_si_001.pdf [file nl4c06400_si_001.pdf]

Supplemental material and supporting information for

Multicomponent magneto-orbital order and magneto-orbitons in monolayer VCl_3

Luigi Camerano[a], Adolfo O. Fumega[b], Gianni Profeta[a,c], Jose L. Lado[b]

[a] Department of Physical and Chemical Sciences, University of L'Aquila, Via Vetoio 67100 L'Aquila, Italy
[b] CNR-SPIN L'Aquila, Via Vetoio 67100 L'Aquila, Italy
[c] Department of Applied Physics, Aalto University, 02150 Espoo, Finland

CONTENTS

I. Methods 2

II. Correlation driven orbital ordering 2

III. Comparison between FO and AFO energies 2

IV. Projected density of states of AFO and FO phases 3

References 4

I. METHODS

Density functional theory calculations were performed using the Vienna ab-initio Simulation Package (VASP) [1, 2], using both the generalized gradient approximation (GGA), in the Perdew-Burke-Ernzerhof (PBE) parametrization for the exchange-correlation functional [3] and local density approximation (LDA). Interactions between electrons and nuclei were described using the projector-augmented wave method. Energy thresholds for the self-consistent calculation was set to 10^{-7} eV and force threshold for geometry optimization 10^{-5} eV Å^{-1}. A plane-wave kinetic energy cutoff of 500 eV was employed for VCl_3. The Brillouin zone was sampled using a 12 × 12 × 1 Gamma-centered Monkhorst-Pack grid. To account for the on-site electron-electron correlation we used the GGA+U. The linear response effective Hubbard term U calculated by Ref. [4] is U = 3.27 eV. The precondition on the on-site d-density matrix was set using the method explained in Refs. [5, 6].

II. CORRELATION DRIVEN ORBITAL ORDERING

In Fig. S1 we report the energies of the converged phases as a function of the Hubbard repulsion U. In the inset an electronic instability driven by the U drives the system in a symmetry broken antiferro-orbital (AFO) phase. As the value of U increases, the metallic $a_{1g}^1 e_g'^1$ solution becomes energetically disfavored compared to both the insulating $e_g'^2$ and the SB-AFO solutions. For any value of $U > 1$ eV, the SB-AFO phase is the ground state.

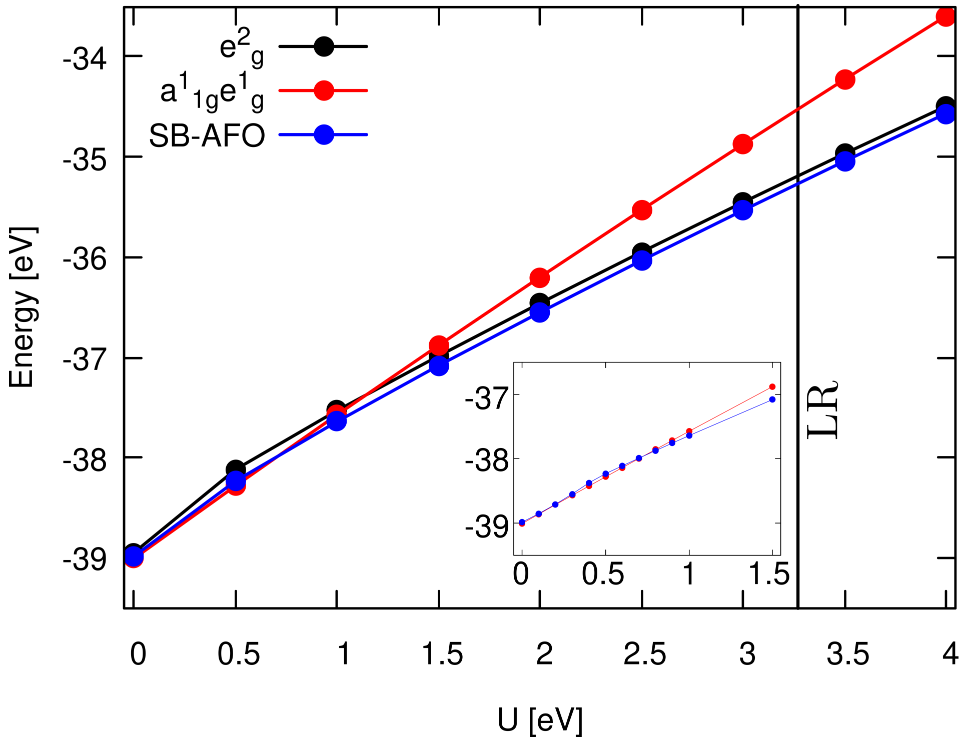

FIG. S1. DFT+U energies of the converged phases as a function of the U. In the inset a zoom of the low U region is reported. The black line at $U = 3.27eV$ corresponds to the linear response (LR) calculated value of the U (see Methods).

III. COMPARISON BETWEEN FO AND AFO ENERGIES

In Fig. S2 we report the energy differences between FO and AFO phase with ferromagnetic spin arrangment. For $a > 6.3$ Åthe AFO phase cannot be converged by first principle calculations.

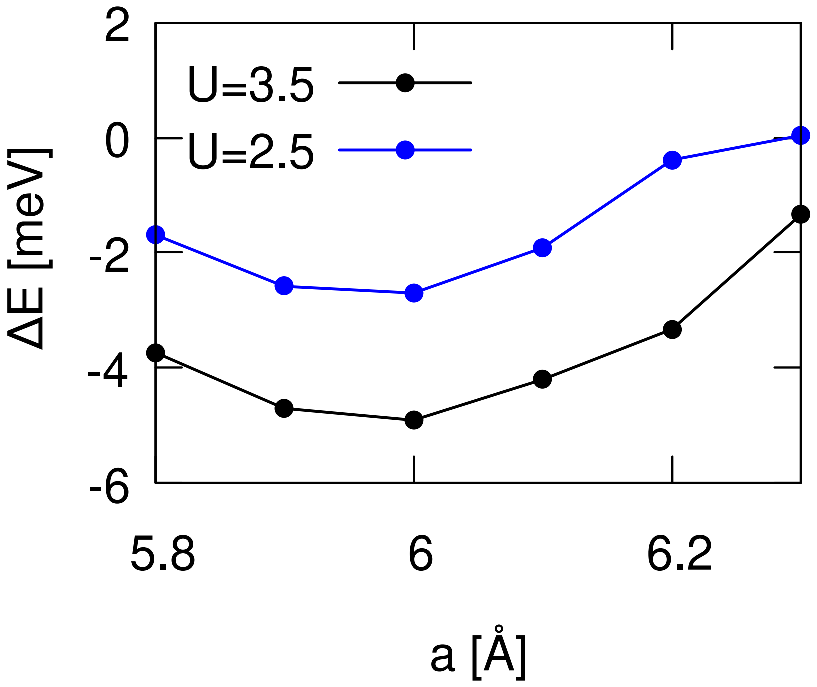

FIG. S2. DFT+U energies of the converged phases as a function of the strain and for different value of the U.

IV. PROJECTED DENSITY OF STATES OF AFO AND FO PHASES

In Fig. S3 the projected density of states (PDOS) of the AFO and FO phases on the vanadium d orbitals is reported. It is worth noting how in the case of the FO phase the on-site PDOS is identical for V_1 and V_2 while in the case of AFO the occupation of the d_{z^2} remains unchanged and the d_{xy} and $d_{x^2-y^2}$ orbitals are almost switched. These orbitals are related with the a_{1g} singlet and the e'_g manifold according to the relation shown in Table S1. The imperfect switching between d_{xy} and $d_{x^2-y^2}$ in the AFO phase may stem from the intricate interactions between different vanadium atoms mediated by the halides in VCl_3. This complexity is fully captured by our first-principles calculations mapping onto model Hamiltonian.

Cubic basis		Trigonal Basis	
Symmetry	Orbitals	Symmetry	Orbitals
t_{2g}	$d_{\bar{x}\bar{y}}$	a_{1g}	d_{z^2}
	$d_{\bar{y}\bar{z}}$	$e'_{g\mp}$	$\frac{2}{\sqrt{6}}d_{xy} + \frac{2}{\sqrt{3}}d_{yz}$
	$d_{\bar{x}\bar{z}}$		$\frac{2}{\sqrt{6}}d_{x^2-y^2} - \frac{2}{\sqrt{3}}d_{xz}$

TABLE S1. Relationships among the atomic d orbitals in the cubic basis ($\bar{x}$, $\bar{y}$, $\bar{z}$) with $\bar{z}$ aligned to the along one of the octhaedral axis and trigonal basis (x,y,z) with z axis aligned to (111) direction.

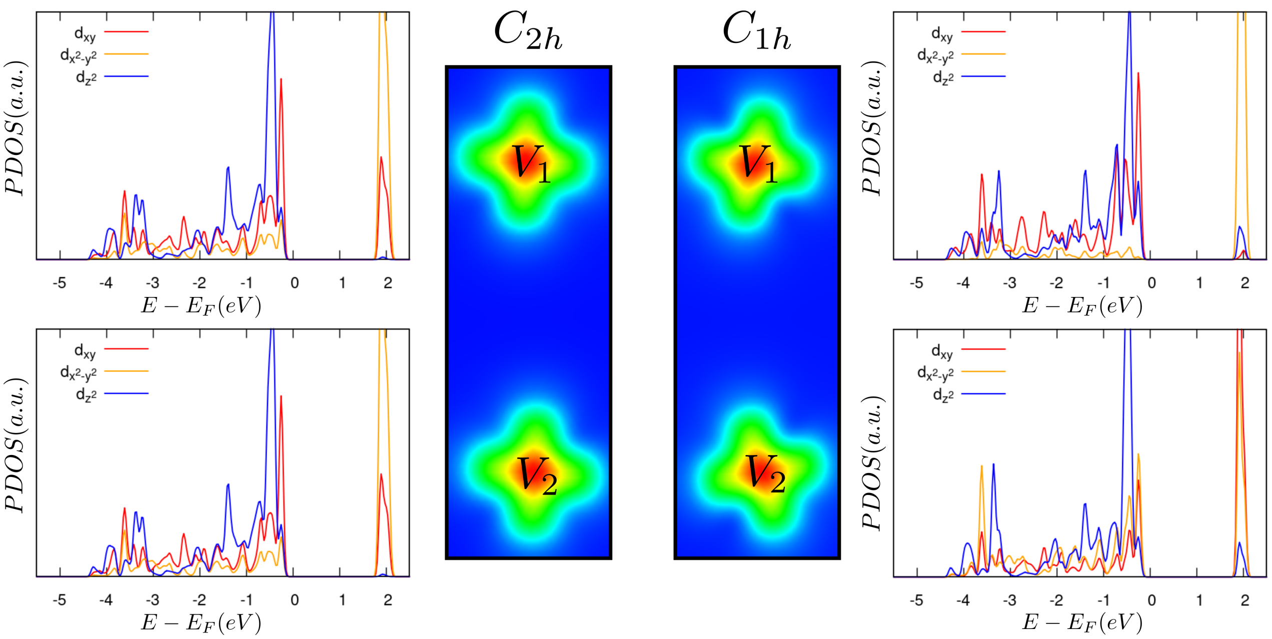

FIG. S3. DFT+U PDOS of the converged phases for the different vanadium sites.

References

[1] G. Kresse and J. Hafner, "Ab initio molecular dynamics for liquid metals," Phys. Rev. B **47**, 558–561 (1993).

[2] G. Kresse and D. Joubert, "From ultrasoft pseudopotentials to the projector augmented-wave method," Phys. Rev. B **59**, 1758–1775 (1999).

[3] John P. Perdew, Kieron Burke, and Matthias Ernzerhof, "Generalized gradient approximation made simple," Phys. Rev. Lett. **77**, 3865–3868 (1996).

[4] Junjie He, Shuangying Ma, Pengbo Lyu, and Petr Nachtigall, "Unusual dirac half-metallicity with intrinsic ferromagnetism in vanadium trihalide monolayers," Journal of Materials Chemistry C **4**, 2518–2526 (2016).

[5] Luigi Camerano and Gianni Profeta, "Symmetry breaking in vanadium trihalides," 2D Materials **11**, 025027 (2024).

[6] Jeremy P. Allen and Graeme W. Watson, "Occupation matrix control of d- and f-electron localisations using dft + u," Phys. Chem. Chem. Phys. **16**, 21016–21031 (2014).
